# 1,5-Bis[1-(4-meth­oxy­phen­yl)ethyl­idene]thio­carbonohydrazide

**DOI:** 10.1107/S1600536811029023

**Published:** 2011-07-23

**Authors:** Xinyu Zhao

**Affiliations:** a School of Petrochemical Engineering, Shenyang University of Technology, Liaoyang 111003, People’s Republic of China

## Abstract

In the title mol­ecule, C_19_H_22_N_4_O_2_S, the two benzene rings form a dihedral angle of 9.16 (13)°. In the crystal, pairs of weak inter­molecular C—H⋯S hydrogen bonds link the mol­ecules into centrosymmetric dimers, which are further linked through weak C—H⋯O inter­actions into sheets parallel to (012).

## Related literature

For related Schiff base derivatives of thio­carbonohydrazide, see: Loncle *et al.* (2004 ▶); Camp *et al.* (2010 ▶). For a related structure, see: Affan *et al.* (2010 ▶).
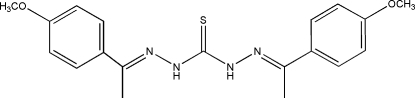


## Experimental

### 

#### Crystal data

C_19_H_22_N_4_O_2_S
*M* *_r_* = 370.47
Monoclinic, 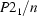

*a* = 7.4225 (6) Å
*b* = 11.4705 (11) Å
*c* = 21.749 (2) Å
β = 91.781 (1)°
*V* = 1850.8 (3) Å^3^
*Z* = 4
Mo *K*α radiation
μ = 0.20 mm^−1^
*T* = 298 K
0.41 × 0.39 × 0.35 mm

#### Data collection

Bruker SMART APEX CCD area-detector diffractometer
Absorption correction: multi-scan (*SADABS*; Sheldrick, 1996 ▶) *T*_min_ = 0.924, *T*_max_ = 0.935
9108 measured reflections
3256 independent reflections
1829 reflections with *I* > 2σ(*I*)
*R*_int_ = 0.050

#### Refinement

*R*[*F* ^2^ > 2σ(*F* ^2^)] = 0.049
*wR*(*F* ^2^) = 0.135
*S* = 1.05
3256 reflections
239 parameters
H-atom parameters constrained
Δρ_max_ = 0.26 e Å^−3^
Δρ_min_ = −0.21 e Å^−3^

### 

Data collection: *SMART* (Bruker, 2007 ▶); cell refinement: *SAINT* (Bruker, 2007 ▶); data reduction: *SAINT*; program(s) used to solve structure: *SHELXS97* (Sheldrick, 2008 ▶); program(s) used to refine structure: *SHELXL97* (Sheldrick, 2008 ▶); molecular graphics: *SHELXTL* (Sheldrick, 2008 ▶); software used to prepare material for publication: *SHELXTL*.

## Supplementary Material

Crystal structure: contains datablock(s) I, global. DOI: 10.1107/S1600536811029023/cv5100sup1.cif

Structure factors: contains datablock(s) I. DOI: 10.1107/S1600536811029023/cv5100Isup2.hkl

Supplementary material file. DOI: 10.1107/S1600536811029023/cv5100Isup3.cml

Additional supplementary materials:  crystallographic information; 3D view; checkCIF report

## Figures and Tables

**Table 1:** Hydrogen-bond geometry (Å, °)

*D*—H⋯*A*	*D*—H	H⋯*A*	*D*⋯*A*	*D*—H⋯*A*
C17—H17⋯O1^i^	0.93	2.59	3.478 (4)	161
C3—H3*A*⋯S1^ii^	0.96	2.90	3.503 (3)	122

Symmetry codes: (i) 
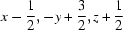
; (ii) 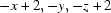.

**Fig. 1.**
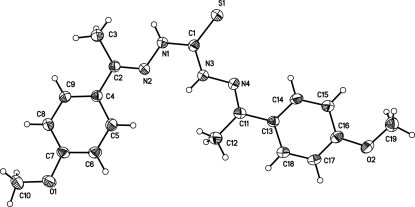
View of (I) showing the atomic numbering and 30% probability displacement ellipsoids.

## supplementary crystallographic information

### Comment

Thiocarbonohydrazide and it's Schiff base derivatives have attracted considerable interest in the chemistry of metal complexes, owing to their wide range of biological activities and catalytical abilities (Loncle *et al.*, 2004; Camp *et al.*, 2010). Herein, we report the crystal structure of the title compound, (I), which is a new derivative of thiocarbonohydrazide.

In (I) (Fig. 1), the bond lengths and angles are normal and correspond to those observed in 1,5-bis[(*E*)-1-(2-hydroxyphenyl)ethylidene] thiocarbonohydrazide monohydrate (Affan *et al.*, 2010). Four N atoms and the C=S are almost coplanar. The N1/N2/C2 plane and the benzene ring C4-C9 form a dihedral angle of 12.20 (3) °. The benzene ring C4-C9 and the benzene ring C13-C18 form a dihedral angle of 9.16 (13) °.

In the crystal structure, weak intermolecular C—H···S hydrogen bonds (Table 1) link the molecules into centrosymmetric dimers, which are further linked through the weak C—H···O interactions (Table 1) into sheets parallel to (012) plane.

### Experimental

4-Methoxylacetophenone (10.0 mmol), 30 ml of ethanol and thiocarbohydrazide (5.0 mmol) were mixed in 50 ml flash. After stirring 3 h at 373 K, the resulting mixture was cooled to room temperature, and recrystalized from ethanol, and afforded the title compound as a crystalline solid.

### Refinement

All H atoms were placed in geometrically idealized positions (N—H 0.86 and C—H 0.93–0.96 Å) and treated as riding on their parent atoms, with *U*_iso_(H) = 1.2–1.5*U*_eq_(C, N).

### Crystal data

**Table:**

C_19_H_22_N_4_O_2_S	*F*(000) = 784
*M**_r_* = 370.47	*D*_x_ = 1.330 Mg m^−^^3^
Monoclinic, *P*2_1_/*n*	Mo *K*α radiation, λ = 0.71073 Å
*a* = 7.4225 (6) Å	Cell parameters from 1954 reflections
*b* = 11.4705 (11) Å	θ = 2.6–21.8°
*c* = 21.749 (2) Å	µ = 0.20 mm^−^^1^
β = 91.781 (1)°	*T* = 298 K
*V* = 1850.8 (3) Å^3^	Block, yellow
*Z* = 4	0.41 × 0.39 × 0.35 mm

### Data collection

**Table:**

Bruker SMART APEX CCD area-detector diffractometer	3256 independent reflections
Radiation source: fine-focus sealed tube	1829 reflections with *I* > 2σ(*I*)
graphite	*R*_int_ = 0.050
φ and ω scans	θ_max_ = 25.0°, θ_min_ = 2.6°
Absorption correction: multi-scan (*SADABS*; Sheldrick, 1996)	*h* = −8→8
*T*_min_ = 0.924, *T*_max_ = 0.935	*k* = −13→13
9108 measured reflections	*l* = −25→25

### Refinement

**Table:**

Refinement on *F*^2^	Primary atom site location: structure-invariant direct methods
Least-squares matrix: full	Secondary atom site location: difference Fourier map
*R*[*F*^2^ > 2σ(*F*^2^)] = 0.049	Hydrogen site location: inferred from neighbouring sites
*wR*(*F*^2^) = 0.135	H-atom parameters constrained
*S* = 1.05	*w* = 1/[σ^2^(*F*_o_^2^) + (0.0435*P*)^2^ + 0.9707*P*] where *P* = (*F*_o_^2^ + 2*F*_c_^2^)/3
3256 reflections	(Δ/σ)_max_ = 0.001
239 parameters	Δρ_max_ = 0.26 e Å^−^^3^
0 restraints	Δρ_min_ = −0.21 e Å^−^^3^

### Special details

**Table:**

Geometry. All e.s.d.'s (except the e.s.d. in the dihedral angle between two l.s. planes) are estimated using the full covariance matrix. The cell e.s.d.'s are taken into account individually in the estimation of e.s.d.'s in distances, angles and torsion angles; correlations between e.s.d.'s in cell parameters are only used when they are defined by crystal symmetry. An approximate (isotropic) treatment of cell e.s.d.'s is used for estimating e.s.d.'s involving l.s. planes.
Refinement. Refinement of *F*^2^ against ALL reflections. The weighted *R*-factor *wR* and goodness of fit *S* are based on *F*^2^, conventional *R*-factors *R* are based on *F*, with *F* set to zero for negative *F*^2^. The threshold expression of *F*^2^ > σ(*F*^2^) is used only for calculating *R*-factors(gt) *etc*. and is not relevant to the choice of reflections for refinement. *R*-factors based on *F*^2^ are statistically about twice as large as those based on *F*, and *R*- factors based on ALL data will be even larger.

### Fractional atomic coordinates and isotropic or equivalent isotropic displacement parameters (Å^2^)

**Table:**

	*x*	*y*	*z*	*U*_iso_*/*U*_eq_	
S1	0.89899 (14)	0.14956 (8)	1.05758 (4)	0.0558 (3)	
N1	0.9376 (4)	0.1693 (2)	0.93818 (11)	0.0464 (7)	
H1	0.9799	0.0995	0.9382	0.056*	
N2	0.9174 (4)	0.2304 (2)	0.88416 (12)	0.0441 (7)	
N3	0.8371 (4)	0.3316 (2)	0.98297 (12)	0.0493 (7)	
H3	0.8383	0.3611	0.9466	0.059*	
N4	0.7809 (4)	0.3991 (2)	1.03058 (12)	0.0462 (7)	
O1	0.8713 (4)	0.46947 (19)	0.62470 (10)	0.0617 (7)	
O2	0.5350 (4)	0.78761 (19)	1.21510 (11)	0.0650 (7)	
C1	0.8904 (4)	0.2201 (3)	0.99129 (14)	0.0416 (8)	
C2	0.9728 (4)	0.1862 (3)	0.83430 (14)	0.0404 (8)	
C3	1.0640 (6)	0.0700 (3)	0.83080 (16)	0.0620 (11)	
H3A	1.1369	0.0577	0.8675	0.093*	
H3B	1.1389	0.0682	0.7956	0.093*	
H3C	0.9746	0.0097	0.8271	0.093*	
C4	0.9453 (4)	0.2574 (3)	0.77845 (14)	0.0397 (8)	
C5	0.8960 (5)	0.3744 (3)	0.78197 (15)	0.0489 (9)	
H5	0.8768	0.4073	0.8203	0.059*	
C6	0.8753 (5)	0.4419 (3)	0.73063 (15)	0.0515 (9)	
H6	0.8451	0.5202	0.7345	0.062*	
C7	0.8987 (5)	0.3951 (3)	0.67283 (15)	0.0456 (8)	
C8	0.9468 (5)	0.2803 (3)	0.66780 (15)	0.0497 (9)	
H8	0.9647	0.2478	0.6293	0.060*	
C9	0.9687 (5)	0.2128 (3)	0.72022 (15)	0.0488 (9)	
H9	1.0002	0.1347	0.7161	0.059*	
C10	0.8880 (5)	0.4244 (3)	0.56465 (15)	0.0622 (10)	
H10A	1.0064	0.3922	0.5605	0.093*	
H10B	0.8695	0.4859	0.5352	0.093*	
H10C	0.7995	0.3646	0.5574	0.093*	
C11	0.7401 (4)	0.5055 (3)	1.01736 (14)	0.0424 (8)	
C12	0.7505 (5)	0.5578 (3)	0.95444 (15)	0.0570 (10)	
H12A	0.8587	0.5319	0.9356	0.085*	
H12B	0.7517	0.6412	0.9577	0.085*	
H12C	0.6476	0.5337	0.9297	0.085*	
C13	0.6849 (4)	0.5776 (3)	1.06957 (14)	0.0393 (8)	
C14	0.7042 (5)	0.5384 (3)	1.12935 (15)	0.0481 (9)	
H14	0.7525	0.4646	1.1364	0.058*	
C15	0.6550 (5)	0.6040 (3)	1.17903 (15)	0.0503 (9)	
H15	0.6707	0.5749	1.2187	0.060*	
C16	0.5819 (5)	0.7137 (3)	1.16945 (16)	0.0486 (9)	
C17	0.5580 (5)	0.7541 (3)	1.11045 (16)	0.0566 (10)	
H17	0.5070	0.8272	1.1036	0.068*	
C18	0.6088 (5)	0.6877 (3)	1.06136 (16)	0.0539 (9)	
H18	0.5919	0.7170	1.0217	0.065*	
C19	0.5427 (5)	0.7484 (3)	1.27698 (16)	0.0628 (10)	
H19A	0.4640	0.6827	1.2812	0.094*	
H19B	0.5052	0.8102	1.3035	0.094*	
H19C	0.6640	0.7260	1.2881	0.094*	

### Atomic displacement parameters (Å^2^)

**Table:**

	*U*^11^	*U*^22^	*U*^33^	*U*^12^	*U*^13^	*U*^23^
S1	0.0754 (7)	0.0489 (5)	0.0435 (5)	0.0089 (5)	0.0060 (4)	0.0056 (4)
N1	0.062 (2)	0.0384 (15)	0.0387 (16)	0.0058 (14)	0.0058 (14)	0.0021 (13)
N2	0.0564 (19)	0.0396 (15)	0.0365 (16)	0.0022 (14)	0.0035 (13)	0.0019 (13)
N3	0.069 (2)	0.0404 (16)	0.0390 (16)	0.0090 (15)	0.0065 (14)	0.0010 (12)
N4	0.0554 (19)	0.0411 (16)	0.0421 (16)	0.0063 (14)	0.0013 (13)	−0.0048 (13)
O1	0.090 (2)	0.0475 (14)	0.0473 (15)	−0.0034 (13)	0.0007 (13)	0.0103 (12)
O2	0.091 (2)	0.0421 (14)	0.0629 (17)	0.0096 (13)	0.0140 (14)	−0.0056 (12)
C1	0.043 (2)	0.0383 (18)	0.044 (2)	−0.0008 (16)	0.0024 (15)	−0.0028 (15)
C2	0.047 (2)	0.0327 (17)	0.0416 (19)	−0.0051 (15)	0.0052 (16)	−0.0004 (14)
C3	0.094 (3)	0.040 (2)	0.053 (2)	0.008 (2)	0.013 (2)	0.0044 (17)
C4	0.043 (2)	0.0341 (17)	0.0418 (19)	−0.0034 (15)	0.0047 (15)	−0.0017 (15)
C5	0.064 (2)	0.0397 (19)	0.043 (2)	−0.0035 (17)	0.0013 (17)	−0.0059 (16)
C6	0.071 (3)	0.0345 (18)	0.049 (2)	−0.0035 (17)	−0.0032 (18)	−0.0012 (17)
C7	0.051 (2)	0.0391 (19)	0.047 (2)	−0.0077 (16)	0.0031 (17)	0.0078 (16)
C8	0.064 (2)	0.047 (2)	0.039 (2)	0.0009 (18)	0.0059 (17)	−0.0002 (16)
C9	0.066 (3)	0.0331 (17)	0.048 (2)	0.0023 (17)	0.0079 (18)	−0.0005 (16)
C10	0.065 (3)	0.076 (3)	0.046 (2)	0.000 (2)	0.0095 (19)	0.0107 (19)
C11	0.041 (2)	0.0394 (19)	0.046 (2)	0.0008 (16)	−0.0027 (15)	0.0039 (15)
C12	0.070 (3)	0.047 (2)	0.053 (2)	0.0007 (19)	−0.0007 (19)	0.0031 (17)
C13	0.040 (2)	0.0346 (17)	0.043 (2)	−0.0029 (15)	−0.0017 (15)	0.0010 (15)
C14	0.055 (2)	0.0325 (17)	0.056 (2)	0.0041 (16)	0.0022 (18)	0.0044 (16)
C15	0.060 (2)	0.0436 (19)	0.048 (2)	0.0039 (18)	0.0044 (18)	0.0051 (16)
C16	0.049 (2)	0.0371 (19)	0.060 (2)	−0.0026 (17)	0.0070 (18)	−0.0017 (17)
C17	0.071 (3)	0.0379 (19)	0.060 (2)	0.0167 (19)	−0.001 (2)	0.0043 (18)
C18	0.068 (3)	0.044 (2)	0.050 (2)	0.0109 (19)	−0.0017 (18)	0.0056 (16)
C19	0.070 (3)	0.061 (2)	0.057 (2)	−0.006 (2)	0.011 (2)	−0.0051 (19)

### Geometric parameters (Å, °)

**Table:**

S1—C1	1.653 (3)	C8—C9	1.384 (4)
N1—C1	1.350 (4)	C8—H8	0.9300
N1—N2	1.372 (3)	C9—H9	0.9300
N1—H1	0.8600	C10—H10A	0.9600
N2—C2	1.277 (4)	C10—H10B	0.9600
N3—C1	1.349 (4)	C10—H10C	0.9600
N3—N4	1.368 (3)	C11—C13	1.473 (4)
N3—H3	0.8600	C11—C12	1.499 (4)
N4—C11	1.288 (4)	C12—H12A	0.9600
O1—C7	1.361 (4)	C12—H12B	0.9600
O1—C10	1.414 (4)	C12—H12C	0.9600
O2—C16	1.359 (4)	C13—C14	1.379 (4)
O2—C19	1.418 (4)	C13—C18	1.393 (4)
C2—C4	1.472 (4)	C14—C15	1.375 (4)
C2—C3	1.498 (4)	C14—H14	0.9300
C3—H3A	0.9600	C15—C16	1.383 (4)
C3—H3B	0.9600	C15—H15	0.9300
C3—H3C	0.9600	C16—C17	1.371 (4)
C4—C9	1.382 (4)	C17—C18	1.374 (4)
C4—C5	1.394 (4)	C17—H17	0.9300
C5—C6	1.364 (4)	C18—H18	0.9300
C5—H5	0.9300	C19—H19A	0.9600
C6—C7	1.383 (4)	C19—H19B	0.9600
C6—H6	0.9300	C19—H19C	0.9600
C7—C8	1.370 (4)		
			
C1—N1—N2	119.2 (3)	O1—C10—H10A	109.5
C1—N1—H1	120.4	O1—C10—H10B	109.5
N2—N1—H1	120.4	H10A—C10—H10B	109.5
C2—N2—N1	119.7 (3)	O1—C10—H10C	109.5
C1—N3—N4	122.0 (3)	H10A—C10—H10C	109.5
C1—N3—H3	119.0	H10B—C10—H10C	109.5
N4—N3—H3	119.0	N4—C11—C13	115.4 (3)
C11—N4—N3	116.3 (3)	N4—C11—C12	124.4 (3)
C7—O1—C10	117.8 (3)	C13—C11—C12	120.1 (3)
C16—O2—C19	119.5 (3)	C11—C12—H12A	109.5
N3—C1—N1	112.2 (3)	C11—C12—H12B	109.5
N3—C1—S1	125.8 (2)	H12A—C12—H12B	109.5
N1—C1—S1	122.1 (2)	C11—C12—H12C	109.5
N2—C2—C4	116.1 (3)	H12A—C12—H12C	109.5
N2—C2—C3	123.7 (3)	H12B—C12—H12C	109.5
C4—C2—C3	120.2 (3)	C14—C13—C18	116.5 (3)
C2—C3—H3A	109.5	C14—C13—C11	121.3 (3)
C2—C3—H3B	109.5	C18—C13—C11	122.1 (3)
H3A—C3—H3B	109.5	C15—C14—C13	122.7 (3)
C2—C3—H3C	109.5	C15—C14—H14	118.7
H3A—C3—H3C	109.5	C13—C14—H14	118.7
H3B—C3—H3C	109.5	C14—C15—C16	119.5 (3)
C9—C4—C5	116.6 (3)	C14—C15—H15	120.3
C9—C4—C2	122.2 (3)	C16—C15—H15	120.3
C5—C4—C2	121.2 (3)	O2—C16—C17	116.4 (3)
C6—C5—C4	121.7 (3)	O2—C16—C15	124.4 (3)
C6—C5—H5	119.2	C17—C16—C15	119.2 (3)
C4—C5—H5	119.2	C16—C17—C18	120.6 (3)
C5—C6—C7	120.6 (3)	C16—C17—H17	119.7
C5—C6—H6	119.7	C18—C17—H17	119.7
C7—C6—H6	119.7	C17—C18—C13	121.5 (3)
O1—C7—C8	125.1 (3)	C17—C18—H18	119.2
O1—C7—C6	115.8 (3)	C13—C18—H18	119.2
C8—C7—C6	119.1 (3)	O2—C19—H19A	109.5
C7—C8—C9	119.8 (3)	O2—C19—H19B	109.5
C7—C8—H8	120.1	H19A—C19—H19B	109.5
C9—C8—H8	120.1	O2—C19—H19C	109.5
C4—C9—C8	122.2 (3)	H19A—C19—H19C	109.5
C4—C9—H9	118.9	H19B—C19—H19C	109.5
C8—C9—H9	118.9		

### Hydrogen-bond geometry (Å, °)

**Table:**

*D*—H···*A*	*D*—H	H···*A*	*D*···*A*	*D*—H···*A*
C17—H17···O1^i^	0.93	2.59	3.478 (4)	161.
C3—H3A···S1^ii^	0.96	2.90	3.503 (3)	122.

Symmetry codes: (i) *x*−1/2, −*y*+3/2, *z*+1/2; (ii) −*x*+2, −*y*, −*z*+2.
